# Commentaries on *the Organon**Read before the New York Homœopathic Union, and proven by experiment upon a little apparatus contrived for the purpose.

**Published:** 1892-06

**Authors:** B. Fincke

**Affiliations:** Brooklyn, N. Y.


### COMMENTARIES ON *THE ORGANON.**

Practical illustration of the homœopathic law by the magnet (§§ 26, 29, 45, 156, 167).

B. Fincke, M. D., Brooklyn, N. Y.

"Like cures like" in Homœopathy and "like repels like" in physics is a paradox which finds its explanation in the third law of motion: action and reaction are equal and directed to contrary sides.

Like symptoms observed from a remedy in health cure like symptoms observed from a disease in sickness. The action of the remedy having similar symptoms as those produced by the action of disease, must necessarily, when administered to the patient, meet the reaction of the life-force as far as it is affected by the disease; and thus the two actions, directed to contrary sides, must equalize each other, provided the remedy be given in *a dose corresponding* to the *dynamic* nature of the life-force. But this is not sufficient, says Hahnemann. The remedy, if similar, will always be stronger than the disease, and must be so prepared that it is just sufficient to overcome the disease, so that the surplus of action will disappear in the restored equilibrium of the life-force. This, then, forms the justification of

---

* Read before the New York Homœopathic Union, and proven by experiment upon a little apparatus contrived for the purpose.

high potencies, which do not act by their physical materiality but by their medicinal force.

Now the word cure in the homœopathic sentence relates to a change of the state of the life-force into its contrary by such a medicinal force. If well, it becomes sick; if sick, well. The word cure, therefore, indicates the result of the conversion of the sick into the healthy state by the homœopathic remedy inasmuch as this is opposed to the pathogenesis, and hence repels and nullifies it in virtue of its symptom-simility. The two sentences practically amount to the same, and may be expressed in a sentence common to both: *like turns like into the contrary, or the action equalizes the opposed action or reaction and the natural state continues.*

This might be exemplified by the action of magnets. Every magnet has a north and south pole. These poles are similar in that both attract iron and both repel like poles, and *vice versâ.* If a magnetic needle is hung up on a silk fibre, it points north and south. This is to represent the life-force in its normal condition.

Suppose we want to explore the action of a remedy and give it to the healthy, the reaction of the life-force will show the action of the remedy in a mutual action, which can be observed in the pathopoëtic series of symptoms called a proving. This is exemplified by the needle when we approach to its north pole the north pole of another magnetic needle called the pathopoëtic needle. It turns the needle into its opposite, for now the south pole points northward. This reversed state of the needle stands for artificial disease or proving. If we remove the pathopoëtic needle, the needle returns to its natural state, pointing north and south just as the life-force after proving a remedy returns to its natural condition. Now in a similar way, as we make our provings, nature produces the diseases by turning the state of health into its contrary, disease, and in this sense the pathopoëtic needle becomes pathogenetic, because natural disease cannot be produced by us, but is generated by nature. But, says Hahnemann, in most cases the life-force is not strong enough to return to its normal condition. Therefore remedies must be applied, which according to the symptom-simility in appropriate dose are able to accomplish a cure.

We assume now that the reversed state of the needle formerly brought out by the pathopoëtic needle signifies the disease produced by nature, the pathogenetic force. What, then, is to be done to restore the normal stand of the needle? According to the magnetic law a pole like the one which has turned the north pole south must be applied to the north pole of the needle now on the opposite side pointing south. But this will only equalize the influence of the opposite pathogenetic needle if it is stronger. In this case the needle turns round again and assumes its normal position in the meridian. The force of the pathogenetic needle is equalized and it is as if it and the pathopoëtic needle were no more in existence. If we in the moment of restoration succeed in removing simultaneously these two needles, the needle acted on continues in its equilibrium as before, and thus represents the perfected cure of the life-force. This actually happens in many cures where the patients recover without aggravations of any kind. The oscillations before the needle comes to rest may be taken for the symptoms.

Hahnemann (section 29) explains this equalization in a manner which amounts to the same thing. For he imagines that the pathopoëtic force produces a reaction in the organism which, being stronger than the opposed pathogenetic force, puts itself in its place, and in accomplishing this substitution its action ceases on account of the short duration dependent upon its infinitesimality. In section 45 he makes this still more distinct when he says: "that as soon as the life-force, disturbed by the preceding disease-potency (pathogenetic force), is seized more powerfully by the stronger dynamic disease-potency (pathopoëtic force), it for this reason remains affected by this alone; hence the previous similar but weaker force, being only a dynamic force without matter, must cease to act furthermore, to act morbidly upon the life-force, consequently, to exist."

It seems the position of Hahnemann in this matter is so well taken that it cannot be controverted. Its exemplification by the magnetic law upon the ground of the third law of motion shows again the justification of his assertion in section 26, that the homœopathic axiom—like cures like—is founded upon a general homœopathic natural law, and as such is a universal law called Homœosis or General Assimilation.

BROOKLYN, N. Y., March 17th, 1892.

